# Serum Copeptin levels in the emergency department predict major clinical outcomes in adult trauma patients

**DOI:** 10.1186/s12873-020-00310-5

**Published:** 2020-02-24

**Authors:** Fulvio Salvo, Francesco Luppi, Davide M. Lucchesi, Simone Canovi, Stefano Franchini, Alessandra Polese, Francesca Santi, Laura Trabucco, Tommaso Fasano, Anna Maria Ferrari

**Affiliations:** 1 Department of Emergency Medicine Azienda USL-IRCCS di Reggio Emilia, via Amendola 2, 42122 Reggio Emilia, Italy; 2 Present address: Respiratory and Critical Care Institute, Cleveland Clinic Abu Dhabi, Abu Dhabi, UAE; 3 Clinical Chemistry and Endocrinology Laboratory, Department of Diagnostic Imaging and Laboratory Medicine, Azienda USL-IRCCS di Reggio Emilia, via Amendola 2, 42122 Reggio Emilia, Italy; 4 Emergency Department, Ospedale San Raffaele, via Olgettina 60, 20132 Milan, Italy

**Keywords:** Copeptin, Vasopressin, Multiple trauma, Trauma severity index, Lactate, Triage

## Abstract

**Background:**

Early prognostication in trauma patients is challenging, but particularly important. We wanted to explore the ability of copeptin, the C-terminal fragment of arginine vasopressin, to identify major trauma, defined as Injury Severity Score (ISS) > 15, in a heterogeneous cohort of trauma patients and to compare its performances with lactate. We also evaluated copeptin performance in predicting other clinical outcomes: mortality, hospital admission, blood transfusion, emergency surgery, and Intensive Care Unit (ICU) admission.

**Methods:**

This single center, pragmatic, prospective observational study was conducted at Arcispedale Santa Maria Nuova, a level II trauma center in Reggio Emilia, Italy. Copeptin determination was obtained on Emergency Department (ED) arrival, together with venous lactate. Different outcomes were measured including ISS, Revised Trauma Score (RTS), hospital and ICU admission, blood transfusion, emergency surgery, and mortality.

**Results:**

One hundred and twenty five adult trauma patients admitted to the ED between June 2017 and March 2018. Copeptin showed a good ability to identify patients with ISS > 15 (AUC 0.819). Similar good performances were recorded also in predicting other outcomes. Copeptin was significantly superior to lactate in identifying patients with ISS > 15 (*P* 0.0015), and in predicting hospital admission (*P* 0.0002) and blood transfusion (*P* 0.016). Comparable results were observed in a subgroup of patients with RTS 7.84.

**Conclusions:**

In a heterogeneous group of trauma patients, a single copeptin determination at the time of ED admission proved to be an accurate biomarker, statistically superior to lactate for the identification of major trauma, hospital admission, and blood transfusion, while no statistical difference was observed for ICU admission and emergency surgery. These results, if confirmed, may support a role for copeptin during early management of trauma patients.

## Background

The introduction of a standardized trauma response system is a milestone in trauma care and is now considered the standard of care in the vast majority of countries [1]. A typical trauma response system consists of a regional integrated network of first responder units and advance life support units, as well as healthcare facilities with different levels of competence, from rural hospitals to specialized trauma centers, all organized in a hub-and-spoke network: the pre-hospital bypass and an effective early triage are crucial steps to avoid admission to a facility not capable to address the needs of a patient, with inevitable loss of time and possible ominous consequences on prognosis.

Severely injured trauma patients, especially those with altered vital signs, do not pose a particular prognostic challenge during early triage and are usually correctly referred to the appropriate facility [2]. This process is not so obvious for trauma patients who initially appear in good and stable conditions, but whose conditions deteriorate rapidly later on. In this grey area management errors are common: overtriage may be considered an option to avoid misplacement of major trauma patients, but it can also lead to negative consequences, such as burdening trauma centers with inappropriate patients, separating patients from their families and communities, or increasing the number of unnecessary transportations [3].

During the last decades different prognostic tools, including both severity scales and biomarkers, have been developed in order to assist the emergency physician in better estimating the burden of injuries of a traumatized patient and identifying major trauma [4]. Some of them are suitable to be used in the pre-hospital setting, such as the Revised Trauma Score (RTS) [5]. Others can only be obtained after admission to the Emergency Department (ED), such as lactate and other biomarkers [6, 7]. There are also other complex prognostic scores, such as the Injury Severity Score (ISS), which can be calculated only after the completion of all the diagnostic procedures, thus having no role in the triage process [8].

Copeptin is the result of the C-terminal cleavage of the arginine-vasopressin (AVP) prohormone and it has emerged as a surrogate marker of AVP, since they are co-secreted by the posterior pituitary gland in an equimolar quantity and, unlike AVP, copeptin has optimal stability ex-vivo [9]. Copeptin measurement has been proposed in the early rule-out of acute myocardial infarction and in the diagnosis of diabetes insipidus, and its prognostic role has been investigated in many different clinical scenarios, including traumatic brain injury, sepsis, and stroke [10–14]. Copeptin determination in traumatic patients has been performed in two main studies so far, and in both of them its level was measured together with AVP [15, 16]. Both studies included highly selected populations with major trauma [15] and traumatic hemorrhagic shock [16]. Copeptin proved again to be a reliable surrogate marker of AVP in these two cohorts, at least during the hyperacute phase. It was able to efficiently predict the need for blood transfusion in one study [16], despite showing no correlation with systolic blood pressure (SBP) in the other [15]. Moreover, AVP did not correlate with mortality and no other prognostic outcomes were assessed [15, 16]. Data regarding copeptin accuracy in predicting trauma severity and other clinical outcomes in unselected trauma patients, especially in comparison with the widely used lactate, are lacking.

## Methods

### Study design, setting, and population

This is a single center, pragmatic, prospective observational study conducted at Arcispedale Santa Maria Nuova IRCCS, a large level II district hospital in Reggio Emilia, Italy. The hospital serves a population of more than 500,000 people and acts as a Level II Trauma Center in an integrated network with five smaller hospitals, which do not routinely admit major trauma patients, and a Level I Trauma Center in Parma, almost 30 km apart. The study duration was 9 months, between June 2017 and March 2018. The study protocol was approved by the institutional ethics committee; the study was conducted in accordance with the Declaration of Helsinki and with the principles of Good Clinical Practice.

All adult trauma patients who arrived at the ED were screened for enrollment. Exclusion criteria were: cardiac arrest at the time of arrival to the ED; known diabetes insipidus or ongoing AVP therapy; evident isolated traumatic brain injury; age less than 18. The final decision whether to enroll a patient was left to the treating emergency physician and trauma team leader: if the patient was perceived as a “potential major trauma” (for example on the basis of the first quick assessment or of the mechanism of injury), a “trauma code” was activated and the patient was included in the study (see Additional file 1 for further details). The aim of the investigators was to avoid a selection bias (i.e. including only patients with overt high burden of injuries) and to include all those trauma patients for which the emergency physician could not immediately rule out major lesions. Informed legal consent was obtained at the time of enrollment from all the patients capable to provide it. If the patient was considered unable to provide it due to his/her medical condition, the legal consent was obtained from a family member or a legal representative.

On admission to the ED, demographic data and vital signs were recorded. A separate blood tube for copeptin analysis was included in the standardized biochemistry panel for trauma patients, routinely collected within few minutes from ED admission, that includes complete blood count, plasma sodium, potassium, chlorine, creatinine, urea nitrogen, glucose, fibrinogen, prothrombin time, activated partial thromboplastin time, serum ethanol, arterial blood gas analysis and plasma venous lactate. The copeptin tube was labeled with an anonymous code and sent to the laboratory. Chest radiograph and Extended Focused Assessment with Sonography in Trauma (E-FAST) were routinely performed in the emergency room on every patient. Further diagnostic and therapeutic strategies were decided by the treating physician on a case-by-case basis following hospital and international guidelines. All trauma team members were blinded to copeptin result.

Patient demographics and clinical variables were collected by the investigators after the completion of trauma workup, through consultation of clinical records on hospital databases. Results of blood tests and of any additional investigation were recorded, as well as surgical diagnostic and therapeutic procedures, and the number of units of blood transfused during the first 48 h. Prehospital information, when available, was also recorded. RTS was calculated using the first available recording of the included parameters after ED admission. ISS was calculated using the results of all the diagnostic procedures performed during the initial evaluation.

Primary objective was the estimation of copeptin accuracy to identify major trauma, defined as ISS > 15. Secondary objectives consisted in evaluating copeptin performance in predicting major clinical outcomes: mortality, hospital admission, blood transfusion, emergency surgery, and ICU admission. Copeptin performances were compared with those of lactate.

### Laboratory methods

Lactate was measured spectrophotometrically through a lactate oxidase-based method on lithium heparin plasma samples using Siemens ADVIA® 1800 Chemistry System. Internal quality controls were performed on a daily basis and results were considered acceptable when within mean ± 2SD.

Serum samples for copeptin were centrifuged and stored at − 20 °C for batch analysis with a commercial immunofluorescence sandwich immunoassay (B.R.A.H.M.S. Copeptin proAVP KRYPTOR, Thermo Scientific). Assay characteristics declared by manufacturer included a 0.7 pmol/L lower detection limit, < 1.08 pmol/L functional sensitivity and < 8% intra-assay (<10% inter-assay) coefficient of variations for concentrations > 4.0 pmol/L.

### Statistical analysis

Diagnostic accuracy of copeptin and venous lactate for ISS > 15 and secondary outcomes was estimated by means of area under Receiver Operating Characteristic curve (ROC, AUC), with associated 95% exact binomial confidence intervals. Different AUCs were compared according to DeLong [17]. For sample size calculation, with statistical power 1-β = 0.8 and significance level α = 0.05, we scheduled to enrol at least 120 patients, expecting lactate and copeptin AUCs around 0.7 and 0.85 respectively, based on previous studies, and anticipating a 30% prevalence of severe injury in our cohort [6, 12, 18]. Differences among subgroups regarding laboratory and clinical data were tested by means of Mann-Whitney U test or Kruskal-Wallis test for quantitative variables and Fisher’s exact test for categorical variables. Spearman’s coefficient of correlation was estimated to assess the relationship between copeptin concentrations and ISS. Statistical analysis was performed using MedCalc, version 12.7 (MedCalc Software, Ostend, Belgium).

## Results

### Patients’ characteristics and types of trauma

Between June 2017 and March 2018 129 patients were selected to participate in the study. Two patients refused enrollment; one patient had diabetes insipidus; one patient had incomplete data recordings. A total of 125 subjects were included in the final analysis. Demographic data, vital signs recordings, and laboratory data are summarized in Table 1.

**Table 1:** Demographic, clinical, and laboratory data of the study cohort

Patient characteristics	All (*n* = 125)	ISS < =15 (*n* = 64)	ISS > 15 (*n* = 61)	P
Age (years)	47 (32–63)	43 (31–60)	56 (35–68)	0.0945
Male sex (n [%])	83 (66.4%)	42 (65.6%)	41 (67.2%)	0.8515
ISS	14 (5–24)	5 (1–9)	25 (21–29)	< 0.0001
GCS	15 (15–15)	15 (15–15)	15 (14–15)	0.0327
Respiratory rate (bpm)	19 (17–22)	18 (16–20)	20 (18–23)	0.0027
Oxygen saturation (%)	98 (95–99)	98 (96–99)	97 (94–99)	0.0249
SBP (mmHg)	129 (113–140)	130 (120–145)	120 (105–139)	0.0001
Heart rate (bpm)	86 (78–98)	80 (76–90)	90 (80–100)	0.0010
WBC (*10^9^/L)	11.5 (9.4–15.7)	10.4 (8.3–12.8)	13.6 (11.1–19.9)	<0.0001
Prothrombin time (ratio)	1.08 (1.04–1.13)	1.06 (1.01–1.09)	1.1 (1.06–1.17)	0.0001
Glucose (mmol/L)	7.0 (5.9–8.6)	6.2 (5.5–7.0)	8.3 (6.9–10.1)	<0.0001
Lactate (mmol/L)	2.05 (1.41–2.89)	1.65 (1.20–2.63)	2.54 (1.69–3.37)	0.0010
Copeptin (pmol/L)	132.0 (39.05–372.38)	58.1 (16.69–142.45)	317.80 (145.58–516.08)	<0.0001
Transfusion (n [%])	18 (14.6%)	1 (1.6%)	17 (28.8%)	<0.0001
Surgery (n [%])	14 (11.4%)	2 (3.1%)	12 (20.3%)	0.0035
Hospitalization (n [%])	108 (87.8%)	50 (78.1%)	58 (98.3%)	0.0006
Hospital length of stay (days)	9 (2–21)	2 (1–8)	18 (11–30)	<0.0001
ICU admission (n [%])	29 (23.6%)	0 (0%)	29 (49.2%)	<0.0001
ICU length of stay (days)	5 (3–11)	0	5 (3–11)	ND
Mortality (n [%])	4 (3.3%)	0 (0%)	4 (6.8%)	0.0536
RTS	7.84 (7.84–7.84)	7.84 (7.84–7.84)	7.84 (7.44–7.84)	0.0001
RTS < 7.84	20 (16.0%)	2 (3.1%)	18 (29.5%)	0.0001
Mechanism of injury				0.7177
Road accident				
Car	61 (48.8%)	32 (50.0%)	29 (47.5%)	
Motorbike	20 (16.0%)	10 (15.6%)	10 (16.4%)	
Bicycle	14 (11.2%)	8 (12.5%)	6 (9.8%)	
Pedestrian	15 (12.0%)	5 (7.8%)	10 (16.4%)	
Precipitation	10 (8.0%)	5 (7.8%)	5 (8.2%)	
Penetrating	1 (0.8%)	1 (1.6%)	0 (0%)	
Other	4 (3.2%)	3 (4.7%)	1 (1.6%)	

Quantitative variables are reported as median (interquartile range); categorical variables are reported as absolute values and rates. Quantitative variables are reported as median (interquartile range); categorical variables are reported as absolute values and rates. *P* values refer to comparisons between ISS > 15 and ISS < =15 subgroups, using Mann-Whitney U test for quantitative variables and Fisher’s exact test for rates. *ISS* Injury severity score, *ICU* Intensive care unit, *RTS* Revised trauma score, *GCS* Glasgow coma scale, *SBP* Systolic blood pressure, *WBC* White blood cells, *bpm* Breaths per minute (respiratory rate) or beats per minute (heart rate)

### Copeptin

**Fig. 1:**
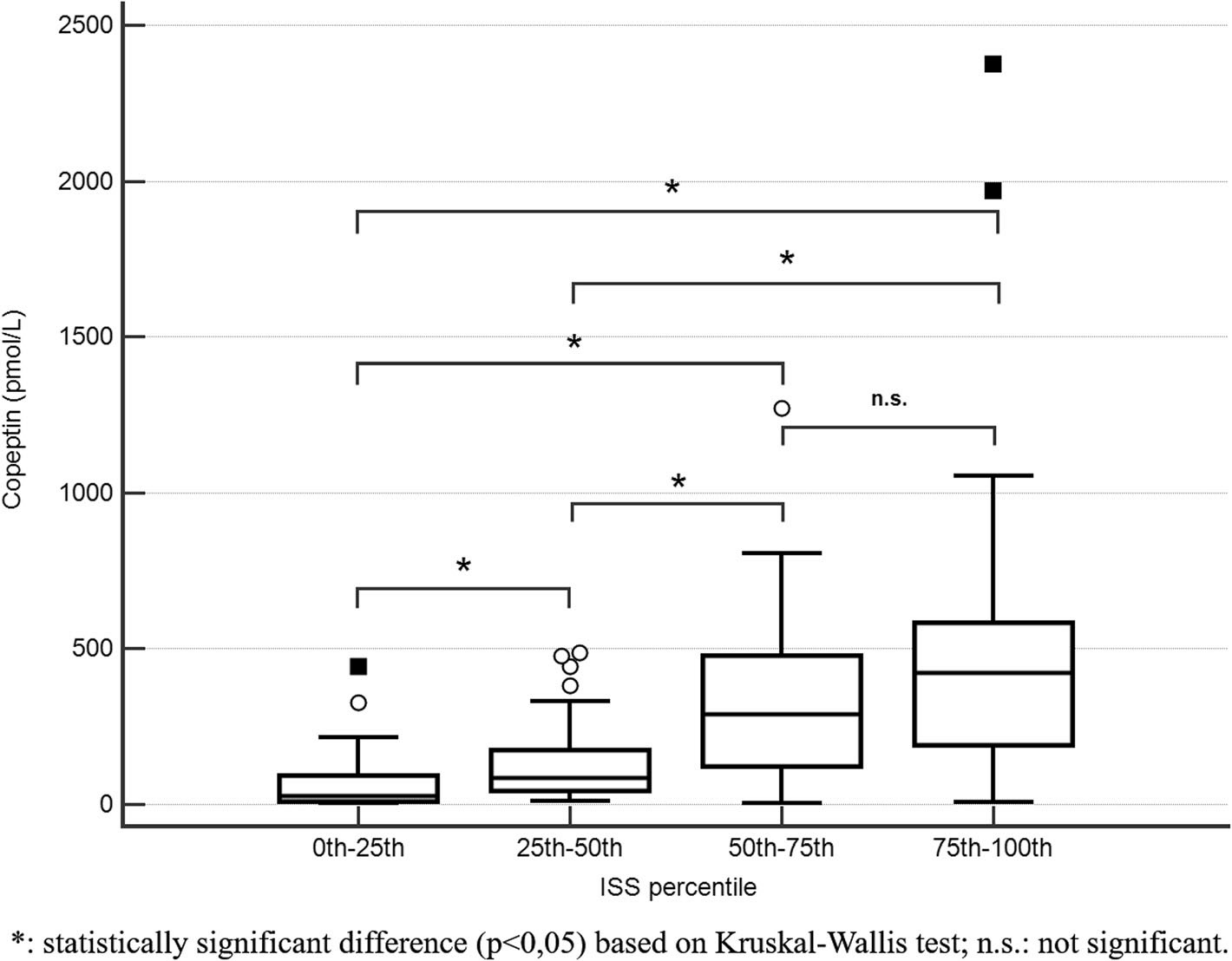
Correlation between copeptin concentration and Injury Severity Scale. *: statistically significant difference (*p* < 0,05) based on Kruskal-Wallis test; n.s.: not significant

Median copeptin concentration in the study population was 132 pmol/L (IQR 39.05–372.38 pmol/L, range 3.4–2373 pmol/L). The upper reference limit for the test is 12 pmol/L, but the optimal decision limit to adopt for prediction of a given clinical outcome in a population of patients with trauma has not been defined yet. Copeptin showed a good performance in predicting ISS > 15, with an AUC of 0.819. Moreover, its concentrations showed a moderate but significant positive correlation with increasing ISS (Spearman’s coefficient of correlation 0.584; 95%CI 0.455–0.689; Fig. 1).

**Fig. 2:**
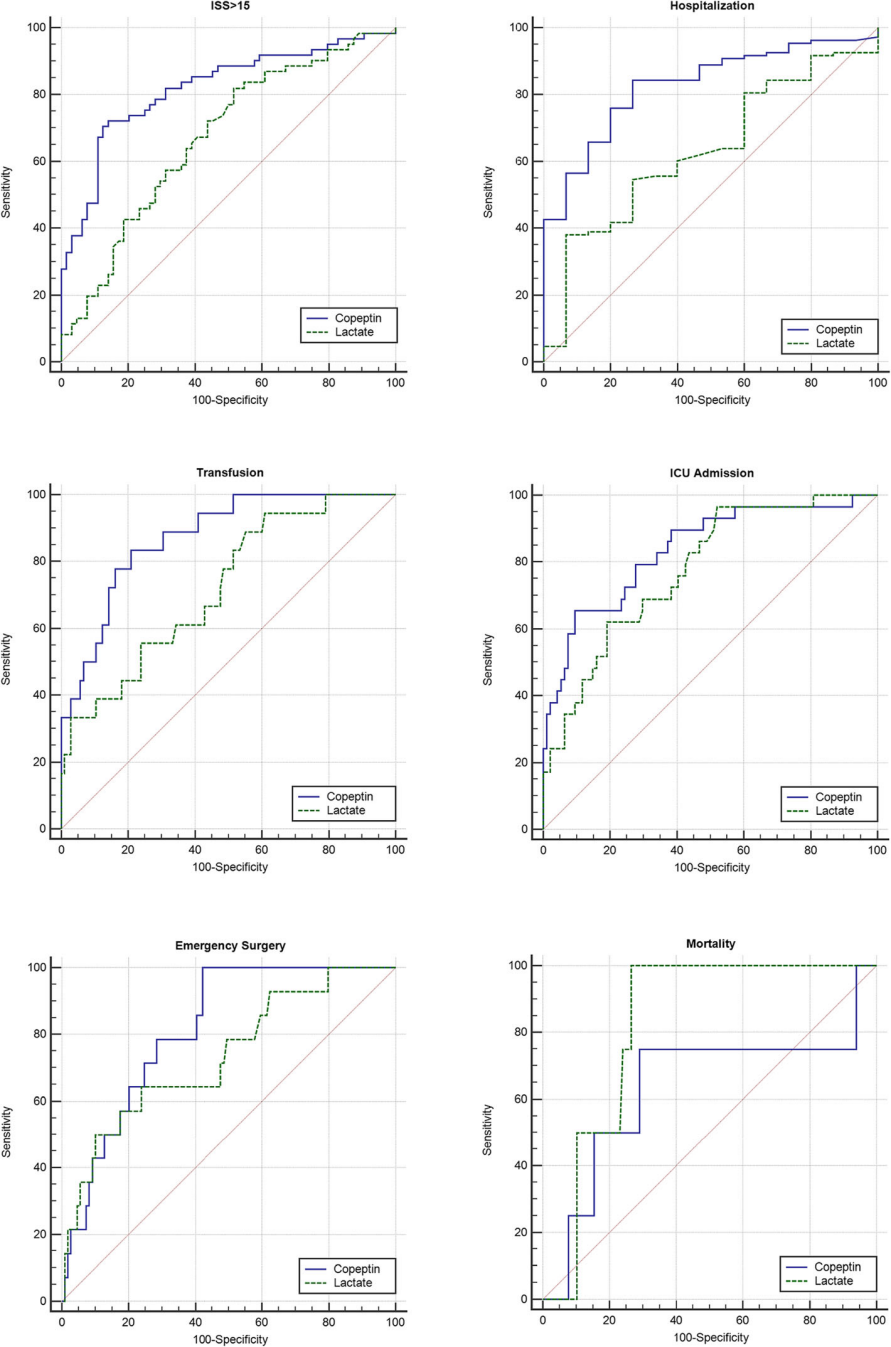
Receiver-operating characteristic curves of copeptin and lactate for primary and secondary endpoints in 125 trauma patients (see text for details)

Copeptin ability to predict secondary outcomes was also good, with AUCs of 0.815 for major surgery, 0.828 for hospitalization, 0.837 for ICU admission and 0.874 for blood transfusion (see Table 2 and Fig. 2). Only the AUC for mortality was a modest 0.635, but the result is conditioned by the low number of deaths registered in our cohort.

**Table 2:** Comparison of AUCs for copeptin and lactate

Outcome	Copeptin AUC (95% CI)	Lactate AUC (95% CI)	*P*-value
A) All trauma patients (n 125)			
ISS > 15	0.819 (0.741–0.882)	0.670 (0.581–0.752)	**0.0015**
Hospital admission	0.828 (0.750–0.890)	0.632 (0.540–0.717)	**0.0002**
ICU admission	0.837 (0.759–0.897)	0.776 (0.692–0.846)	0.2540
Major Surgery	0.815 (0.735–0.879)	0.735 (0.647–0.810)	0.2803
Hemotransfusion	0.874 (0.802–0.927)	0.722 (0.634–0.799)	**0.0164**
Mortality	0.635 (0.542–0.720)	0.824 (0.744–0.887)	0.3914
B) Patients with RTS 7.84 (n 105)			
ISS > 15	0.810 (0.722–0.880)	0.617 (0.517–0.710)	**0.0003**
Hospital admission	0.806 (0.716–0.877)	0.589 (0.488–0.685)	**0.0002**
ICU admission	0.846 (0.761–0.910)	0.741 (0.645–0.822)	0.0882
Major Surgery	0.843 (0.758–0.907)	0.679 (0.580–0.768)	0.0872
Hemotransfusion	0.879 (0.800–0.935)	0.678 (0.579–0.767)	**0.0130**

Comparison of AUCs of copeptin and lactate for the different endpoints in the entire cohort (A) and in the subgroup with RTS = 7.84 (B). ISS: injury severity scale; ICU: intensive care unit; RTS: revised trauma score

### Venous lactate

Median venous lactate concentration in the study population was 2.05 mmol/L (IQR 1.41–2.89 mmol/L), with 51.2% of results above the upper reference limit for the test (2 mmol/L). Lactate AUC for prediction of ISS > 15 resulted in a modest 0.670. The widely used upper reference limit for plasma venous lactate showed only moderate sensitivity (64%) and specificity (63%) in predicting ISS > 15. Adopting the cut-off that maximizes diagnostic accuracy (1.61 mmol/L) increased sensitivity to 82%, but decreased specificity to 48%: overall diagnostic accuracy was nonetheless slightly improved (Youden’s statistics J 0.3 vs 0.27). See Table 3 for reference.

**Table 3:** Relative measures of diagnostic accuracy for different outcomes

Biomarker	Cut-off		Primary outcome ISS > 15			
			Sensitivity (95%CI)	Specificity (95%CI)	+LR (95%CI)	-LR (95%CI)
Lactate	URL	2 mmol/L	0.64 (0.51–0.76)	0.63 (0.50–0.74)	1.7 (1.2–2.5)	0.58 (0.4–0.8)
	MYI	1.61 mmol/L	0.82 (0.70–0.91)	0.48 (0.36–0.61)	1.59 (1.2–2.1)	0.37 (0.2–0.7)
	95% Sens	0.80 mmol/L	N/A	0.14 (0.00–0.28)	1.11 (1.0–1.2)	0.35 (0.10–1.2)
Copeptin	MYI	189 pmol/L	0.72 (0.59–0.83)	0.86 (0.75–0.93)	5.13 (2.7–9.6)	0.32 (0.2–0.5)
	95% Sens	12.4 pmol/L	N/A	0.20 (0.51–0.48)	1.19 (1.0–1.4)	0.24 (0.07–0.8)
Secondary outcome hospital admission						
			Sensitivity (95%CI)	Specificity (95%CI)	+LR (95%CI)	-LR (95%CI)
Lactate	URL	2 mmol/L	0.53 (0.43–0.63)	0.73 (0.45–0.92)	1.98 (0.8–4.7)	0.64 (0.4–0.9)
	MYI	2.60 mmol/L	0.38 (0.29–0.48)	0.93 (0.68–0.99)	5.69 (0.8–38.4)	0.66 (0.5–0.8)
	95% Sens	0.75 mmol/L	N/A	0.13 (0.02–0.41)	1.06 (0.9–1.3)	0.59 (0.1–2.5)
Copeptin	MYI	31 pmol/L	0.84 (0.76–0.91)	0.73 (0.45–0.92)	3.16 (1.4–7.3)	0.21 (0.1–0.4)
	95% Sens	8.1 pmol/L	N/A	0.27 (0.00–0.60)	1.29 (0.9–1.8)	0.21 (0.07–0.7)
Secondary outcome blood transfusion						
			Sensitivity (95%CI)	Specificity (95%CI)	+LR (95%CI)	-LR (95%CI)
Lactate	URL	2 mmol/L	0.67 (0.41–0.87)	0.53 (0.43–0.63)	1.43 (1.0–2.1)	0.63 (0.3–1.2)
	MYI	1.67 mmol/L	0.89 (0.65–0.99)	0.45 (0.35–0.55)	1.61 (1.3–2.0)	0.25 (0.07–0.9)
	95% Sens	1.19 mmol/L	N/A	0.21 (0.08–0.42)	1.19 (1.0–1.4)	0.27 (0.04–1.8)
Copeptin	MYI	291 pmol/L	0.83 (0.59–0.96)	0.79 (0.70–0.86)	3.98 (2.6–6.1)	0.21 (0.07–0.6)
	95% Sens	94.4 pmol/L	N/A	0.49 (0.35–0.64)	1.84 (1.5–2.3)	0.11 (0.02–0.8)
Secondary outcome ICU admission						
			Sensitivity (95%CI)	Specificity (95%CI)	+LR (95%CI)	-LR (95%CI)
Lactate	URL	2 mmol/L	0.76 (0.57–0.90)	0.59 (0.48–0.69)	1.83 (1.3–2.5)	0.41 (0.2–0.8)
	MYI	1.64 mmol/L	0.97 (0.82–0.99)	0.48 (0.38–0.58)	1.85 (1.5–2.3)	0.07 (0.01–0.5)
	95% Sens	1.65 mmol/L	N/A	0.48 (0.17–0.64)	1.85 (1.5–2.3)	0.07 (0.01–0.5)
Copeptin	MYI	400 pmol/L	0.66 (0.46–0.82)	0.90 (0.83–0.96)	6.84 (3.5–13.4)	0.38 (0.2–0.6)
	95% Sens	61.5 pmol/L	N/A	0.43 (0.03–0.67)	1.68 (1.4–2.0)	0.08 (0.01–0.6)
Secondary outcome emergency surgery						
			Sensitivity (95%CI)	Specificity (95%CI)	+LR (95%CI)	-LR (95%CI)
Lactate	URL	2 mmol/L	0.64 (0.35–0.87)	0.52 (0.43–0.62)	1.35 (0.9–2.1)	0.68 (0.3–1.4)
	MYI	2.78 mmol/L	0.64 (0.35–0.87)	0.76 (0.67–0.84)	2.7 (1.6–4.5)	0.47 (0.2–1.0)
	95% Sens	1.18 mmol/L	N/A	0.20 (0.08–0.40)	1.16 (1.0–1.4)	0.35 (0.05–2.4)
Copeptin	MYI	132 pmol/L	1.00 (0.77–1.00)	0.58 (0.48–0.67)	2.37 (1.9–3.0)	0
	95% Sens	146 pmol/L	N/A	0.58 (0.47–0.67)	2.2 (1.7–2.9)	0.12 (0.02–0.8)

Relative measures of diagnostic accuracy (sensitivity, specificity, likelihood ratios) for different cut-offs of lactate and copeptin for primary and secondary outcomes are reported: the limit associated with maximum value of Youden Index and one with high (95%) sensitivity are shown; for lactate, also the upper reference limit (2 mmol/L) is reported. Youden’s J statistics summarizes the diagnostic performance of a test relative to a given cut-off: its value ranges from 0 for non-informative tests to 1 for perfect tests. Cut-off values are in mmol/L (lactate) and pmol/L (copeptin). *URL* Upper reference limit, *MYI* Maximum Youden Index. *95%Sens* Sensitivity of 95%, *CI* Confidence intervals, *+LR* Positive likelihood ratio, *-LR* Negative likelihood ratio, *N/A* Not applicable

The analysis of lactate performance for secondary outcomes also showed a modest to fair predictive capacity. In predicting hospitalization lactate AUC was 0.632. For ICU admission AUC was 0.776. In predicting major emergency surgery AUC was 0.735, whereas for blood transfusion AUC was 0.722. The best lactate AUC estimate was obtained for mortality with a good 0.824 (see Table 2).

### Copeptin vs lactate

Copeptin performed significantly better than venous lactate in predicting major trauma (*P* 0.0015) and was also statistically superior in predicting hospital admission (*P* 0.0002), and blood transfusion (*P* 0.0164). No statistical difference between copeptin and lactate was observed in predicting ICU admission (*P* 0.25) and emergency surgery (*P* 0.28). Also no relevant difference was seen for mortality (*P* 0.39), but the very low death rate observed in our population hampers any further discussion on this outcome (see Fig. 2**,** Table 2).

### Sub-group analysis

In order to explore copeptin diagnostic accuracy in patients without overt initial physiologic derangement, we focused on subjects with RTS 7.84. The weighted form of RTS is calculated as 0.9368*(GSC*c*) + 0.7326*(SBP*c*) + 0.2908*(RR*c*), where *c* is the coded value for each clinical parameter (range 0–4, with 4 corresponding to the normal value). The highest possible score is 7.84, which reflects the absence of relevant alteration in the included parameters (i.e. GCS ≥ 13; SBP ≥ 90; respiratory rate 10–29) and identifies those patients with low risk of adverse outcome [5]. We compared diagnostic accuracy of copeptin and venous lactate in this subgroup of 105 patients. The results confirmed those obtained in the entire study population. Copeptin proved again to be significantly superior to venous lactate in identifying patients with ISS > 15 (AUC 0.810 vs 0.617, *P* 0.0003). Superiority was also confirmed for the secondary outcomes hospitalization (AUC 0.806 vs 0.589, *P* 0.0002) and blood transfusion (AUC 0.879 vs 0.678, *P* 0.013). No statistical difference was observed in emergency surgery (AUC 0.843 vs 0.679, *P* 0.087) and ICU admission (AUC 0.846 vs 0.741, *P* 0.088), despite a trend toward superiority of copeptin (see Table 2). Analysis for mortality was not performed, considering the low prevalence of this outcome in the sub-group (n 2).

## Discussion

In our heterogeneous group of trauma patients, a single copeptin determination at the time of ED admission proved to be an accurate biomarker for the identification of major trauma and a reliable predictor of subsequent hospital admission, emergency surgery, blood transfusion, and ICU admission. Copeptin was superior to lactate in identifying patients with ISS > 15, as well as those who needed hospital admission and blood transfusion. Moreover, in the grey area represented by trauma patients without an overt derangement of physiologic parameters and with a RTS score of 7.84, copeptin was again superior to lactate in predicting major trauma, need for blood transfusion, and hospital admission.

Severe trauma is a complex syndrome encompassing physical damages to multiple organs and tissues, and the physiological reactions to them, including neuroendocrine, metabolic, hemocoagulatory, inflammatory, and immune responses [19–22]. Neuroendocrine response to multiple trauma is complex and far to be completely understood. Only few studies in the literature assessed AVP and/or copeptin in trauma patients [15, 16, 23]. Increased levels can be detected as early as 20 min after trauma, probably even earlier [23]. AVP and copeptin levels proved to be clearly correlated in trauma patients, confirming once more the role of copeptin as a surrogate marker for AVP [15]. Moreover, in a cohort of patients with severe trauma, AVP levels were significantly higher in patients with ISS > 15 compared to those with ISS < 15 [23]. Interestingly AVP did not show any correlation with SBP in patients with trauma, suggesting that, in trauma patients, a drop in SBP may not be the only trigger to AVP release, and/or that AVP secretion may be highly effective in sustaining hemodynamic stability, at least in a first compensated phase [16]. Conversely, in that same study, copeptin and AVP levels were good predictors for the need of transfusions [23]. This was confirmed in another study of severely injured patients with trauma-related hemorrhagic shock, where copeptin showed the best predictive value compared to AVP and lactate (AUC 0.87 vs 0.81 vs 0.79) [16]. These findings were reproduced in our study, where copeptin performed better than lactate in predicting any need of blood transfusion.

Our study is the first to investigate the prognostic ability of copeptin in unselected patients with potential major trauma. In contrast to all the previous studies in this field, which placed a severity criterion at inclusion checkpoint (e.g. ISS > 15 or SBP < 90 mmHg), we decided to leave the final decision to enroll a trauma patient to the attending physician: this pragmatic approach recreates the real-life situation in which a screening tool for prognostic stratification is needed the most [15, 16]. Moreover, considering the lack of correlation between copeptin and blood pressure, including only hypotensive patients would have potentially excluded from the analysis a significant amount of cases with high copeptin levels [16].

In line with the results of previous studies, in our cohort the levels of copeptin were far higher than those commonly found in healthy volunteers [15, 24]. This was true even in the vast majority of patients with minor or negligible injuries. It is evident that stimuli other than increased plasma osmolality or decreased blood volume are responsible for AVP and copeptin release also in this subset of patients. Psychological stress has been shown to increase circulating levels of copeptin, but to an extent much less pronounced [24]. Nevertheless, it is possible that the intense stress experienced by a victim of trauma may trigger a more significant release of AVP from the neurohypophysis, thus increasing the mean copeptin level found in our patients. In light of this, it is advisable that thresholds different from the upper normal limit should be considered in this particular setting.

Prognostication in trauma is extremely challenging: high burden of injuries in multiple sites may not pose an imminent threat to patient’s life, whereas a single lesion to a core organ (for instance central nervous system, great vessels, heart) may prove to be fatal even in the setting of the best available hospital care. A good triage tool should keep the rate of overtriage between 25 and 50% while reducing undertriage to less than 5% [25, 26]. Selected trauma patients first evaluated in a non-tertiary trauma center benefit from being transferred to a level I or level II trauma center: a timely selection of those patients represents one of the main targets of the early management phase [27]. Early lactate is commonly used for prognostic stratification in the ED, but even if it proved to be a good predictor of mortality in multiple trauma patients, it falls short in predicting major trauma and other related outcomes. For instance, in a study by Regnier and colleagues lactate AUC for ISS > 15 was 0.61, while Paladino and co-authors found an AUC of 0.64 when lactate was used to identify major trauma in patients with normal vital signs, both results comparable to our study and definitely lower than 0.82 of copeptin AUC [18, 28].

Recent experimental studies in rat models, documented that acute hypoxia leads to an early and marked increase in copeptin levels, even after only 5 min of hypoxic ventilation [29, 30]. Another study on copeptin cord blood concentrations in neonates also showed that, despite copeptin being strongly correlated to birth acidosis, lactate, and asphyxia (with the highest level found in neonates with asphyxia), no association was found between copeptin and arterial hypotension [31]. Thus, it is plausible that also hypoperfusion could act as a relevant additive stimulus to AVP release.

Several studies suggest that increased copeptin serum concentrations can be detected in chronic kidney disease and that its level may predict future development of chronic kidney failure in a population free from kidney disease at baseline [32, 33]. A correlation between copeptin and creatinine levels was found also in severely-ill patients with acute kidney injury, even if evidence is still scarce [34]. Looking at our data, while we confirm that a weak but positive correlation exists even in our trauma cohort between these two biomarkers (Spearman’s rho 0.297; 95%CI 0.129–0.450, *P* = 0.0008), we found copeptin predictive value to be significantly superior to that of creatinine for all primary and secondary outcomes of the study (*P* < 0.05 for all pairwise ROC curve comparisons, data not shown).

Our data and studies in the literature suggest the increased copeptin and creatinine concentrations in adult trauma patients might share only some pathophysiological stimuli (e.g. hypoperfusion), while copeptin may possibly contribute to further development of acute kidney failure by enhancing endothelial dysfunction and increasing systemic vascular tone [34].

From a metabolic point of view, the accumulation of lactate, a final byproduct of hypoxic cellular metabolism, may represent the downstream result of the complex chain of events that links physical injury to tissue hypoperfusion and hypoxygenation. AVP constitutes one of the first responses of the organism to the traumatic event and its physiologic effects actively counteract the drop in intravascular volume and the consequent development of tissue hypoxia. Its use in hemorrhagic shock was associated with increased survival in animal studies and in several case reports on humans [35, 36]. Although the studies on the relationships between AVP, hypoxia and hypoperfusion are at their early stages, it is intriguing to consider that an early responsiveness of AVP to hypoperfusion (in addition to the other well-known AVP-triggering stimuli) would explain both the stronger correlation of copeptin with the need for transfusion than with hemodynamic parameters reported in previous studies, and its high reliability, even higher than that of lactate, in predicting the clinical course of patient lacking overt initial physiologic derangement that we observed [23]. It is fascinating to speculate that in the challenging situation of early management of trauma patients copeptin may support the clinician in identifying occult hemorrhage before a drop in SBP or in hemoglobin level. This may be particularly useful also from a regional trauma network perspective, where for example the detection of an elevated level of copeptin in a trauma patient with normal vital signs may assist the physician in the decision to transfer the patient from a peripheral hospital without availability of advanced imaging to a higher level trauma center. Nevertheless, with its widespread availability and minimal cost of point-of-care testing, lactate is still an easier to use biomarker in trauma patient. It is anyway worth mentioning that copeptin can be promptly detected by several immunoassay detection methods, and it is now increasingly available in clinical laboratories.

Our study has indeed some limitations. First, it is a single center study performed in a level II trauma center: this means that we missed some of the most severe patients, referred to the level I trauma center directly from the field, as well as some of the less severe ones, possibly treated in the small hospitals of the area. Second, prevalence of penetrating injuries is far less common in our cohort compared to other studies, a factor that could have influenced the distribution of trauma severity and the diagnostic performance of copeptin in this specific cohort. Third, it was not possible to recollect all the data about prehospital management, thus hampering the possibility to ascertain the effect of volume replacement with crystalloids on copeptin levels on ED admission. Finally, the low mortality prevents any possible conclusion on this secondary endpoint. Considering all these factors, our results should be externally validated in other studies.

## Conclusions

In trauma patients without an overt life-threatening injury, copeptin may represent an important aid to the physician, assisting the decision on further management such as more accurate diagnostic tests, prolonged observation, or transfer to a higher level facility. Further research on copeptin in patients with trauma may provide additional evidence to support its use as a prognostic stratification tool, but it might also help in clarifying the complex neuroendocrine response to trauma, possibly providing interesting new pathophysiological insights and therapeutic targets.

## Supplementary information

**Additional file 1.** Supplementary Materials and Methods

## Data Availability

The datasets used and/or analyzed during the current study are available from the corresponding author on reasonable request.

## Abbreviations

AUC: Area under the (ROC) curve
AVP: Arginine vasopressin
ED: Emergency department
E-FAST: Extended focused assessment with sonography for trauma
GCS: Glasgow coma scale
ICU: Intensive care unit
ISS: Injury severity score
ROC: Receiver operating characteristic
RTS: Revised trauma score
SBP: Systolic blood pressure
