# Supplementary material for: Serum Copeptin levels in the emergency department predict major clinical outcomes in adult trauma patients
Source: BMC Emerg Med. 2020 Feb 24;20:14. doi: 10.1186/s12873-020-00310-5 (PMC7041089; doi:10.1186/s12873-020-00310-5)
Supplement: Supplementary file 1 — Additional file 1. Supplementary Materials and Methods [file 12873_2020_310_MOESM1_ESM.docx]

**Supplementary Materials and Methods**

*Study setting: emergency response system organization.*

Arcispedale Santa Maria Nuova is a level II trauma center located in Reggio Emilia. The hospital serves a population of more than 500000 people and it acts as a Level II Trauma Center in an integrated network with five smaller hospitals, which do not routinely admit major trauma patients, and a Level I Trauma Center in Parma, almost 30 km apart. While severe trauma cases from the city of Reggio Emilia are usually referred to Arcispedale Santa Maria Nuova, patients from the countryside can be transported by helicopter directly to the level I trauma center in Parma, depending on the estimated transport time and on the on-site assessment. In line with the national organization of the prehospital emergency system, there are different types of prehospital units on the field. In particular, a mobile resuscitation unit with an emergency physician on board is dispatched on the scene of most severe cases.

*Management of patients in the ED and study protocol*

All patients were managed in accordance with the Hospital Trauma Protocol, based on current international and regional guidelines.

A trauma code is activated after the first assessment by the treating emergency physician and trauma team leader and involves a standardized series of diagnostic procedures. The biochemistry panel for trauma code patients includes complete blood count, plasma sodium, potassium, chlorine, creatinine, urea nitrogen, glucose, fibrinogen, prothrombin time, activated partial thromboplastin time, serum ethanol, arterial blood gas analysis and plasma venous lactate.

Chest radiograph and Extended Focused Assessment with Sonography in Trauma (E-FAST) were routinely performed in the emergency room on every patient. The classic trauma team composition in our institution is: an emergency physician, an emergency medicine resident, three emergency nurses, one healthcare worker, and readily available critical care physician, emergency surgeon, and orthopedic surgeon.

The copeptin tube was labeled with an anonymous code and sent to the laboratory.

Patient demographics and clinical variables were collected by the investigators after the completion of trauma workup, through consultation of clinical records on hospital databases. Results of blood tests and of any additional investigation were recorded, as well as surgical diagnostic and therapeutic procedures, and the number of units of blood transfused during the first 48 hours. Prehospital information, when available, was also recorded. RTS was calculated using the first available recording of the included parameters after ED admission. ISS was calculated using the results of all the diagnostic procedures performed during the initial evaluation.

*Description of outcomes*

Hospital admission: any admission to a hospital ward for >24 hours.

ICU admission: any admission to the ICU directly from the ED or after an emergency surgical procedure.

Emergency surgery: any surgical procedures considered life-saving and undeferrable, including abdominal surgical procedures, thoracic surgical procedures, neurosurgical procedures, and orthopedic procedures with simultaneous hemorrhage control, but excluding orthopedic procedures on distal bones such as bone fixation, fracture reduction or other minor procedures.

Blood transfusion: any transfusion of packed red blood cells (+/- plasma) occurring in the first 48 hours and clearly related to the traumatic culprit. Platelet transfusions were not included (and did not actually occur in our cohort). Any transfusion performed after 48 hours from hospital admission was not included.

*Statistical analysis*

In the analysis of the main outcome (ISS>15) all 125 subjects were included. For the secondary outcomes hospital admission, ICU admission, blood transfusions, and emergency surgery two patients who died in the ED were excluded. For the outcome mortality four patients who were transferred to other facilities or who left the hospital against physician advice within the first 48 hours were excluded.
